# Dementia Praecox

**Published:** 1914-05

**Authors:** 


Dementia Praecox.—Bayard Holmes lays stress on the fact that a positive laboratory diagnosis of this disease, from which probably 60 per cent of asylum inmates are suffering, can now be made by means of Abderhalden's defensive ferment reaction, Fauser having demonstrated a degenerative process of the testicle or ovary in this disease which can be recognized by the Abderhalden procedure. He also quotes approvingly Lundvall's treatment of the disease, which is said to cure 30 per cent or more and consists in injecting subcutaneously, when the blood has shown a polycythemia and leucopenia, two to twenty cc. of a solution made up of sodium nucleate, 100 parts; arsenic trioxide, 0.05; hetol (sodium cinnamate), 10; and distilled water, 400. This should be sterilized before use and administered warm. The dose should be repeated as soon as the resulting leucocytosis has subsided, and the treatment continued over a long time.

